# Effects of domestication on responses of chickens and red junglefowl to conspecific calls: A pilot study

**DOI:** 10.1371/journal.pone.0279553

**Published:** 2022-12-30

**Authors:** Vitor Hugo Bessa Ferreira, Mylène Dutour, Rebecca Oscarsson, Johanna Gjøen, Per Jensen

**Affiliations:** 1 IFM Biology, AVIAN Behavioural Genomics and Physiology Group, Linköping University, Linköping, Sweden; 2 School of Biological Sciences, University of Western Australia, Crawley, WA, Australia; Tokai University School of Medicine, JAPAN

## Abstract

Beyond physical and zootechnical characteristics, the process of animal domestication has also altered how domesticated individuals, compared to their wild counterparts, perceive, process, and interact with their environment. Little is known, however, on whether and how domestication altered the perception of conspecific calls on both domesticated and wild breeds. In the present work, we compared the vigilance behavior of domestic and captive-born wild fowl following the playback of chicken alarm calls and contentment calls (control). The playback tests were performed on four different breeds/lines. We first compared the behavioral reaction of domesticated White Leghorn (WL, a breed selected for egg production) and Red Junglefowl (RJF) hens (ancestor of domestic chickens). We also compared the behavior of Red Junglefowl hens selected for high or low fear of humans (RJF HF and RJF LF, respectively), a proxy to investigate early effects of domestication. Contrary to our expectations, no breed/line reacted accordingly to the calls, as the increase in vigilance behavior after the playback calls was similar for both alarm and contentment calls. Although no call discrimination differences were found, breeds did differ on how they reacted/habituated to the calls. Overall, WL were more vigilant than RJF, and birds from the RJF LF line decreased their vigilance over testing days, while this was not the case for the RJF HF line. These results suggest that birds under commercial-like conditions are unable to discriminate between alarm and contentment calls. Interestingly, domestication and selection for low fear of humans may have altered how birds react to vocal stimuli. It is important to consider that farmed animals may interpret and be affected by the vocalizations of their conspecifics in unexpected ways, which warrants further investigation.

## Introduction

In many animals, acoustic communication plays a central role in a variety of behavioral contexts such as mate selection, territorial defense, predator avoidance, group cohesion and foraging [1]. Perceptual and communicative abilities of species may have been altered throughout domestication [2, 3]. Indeed, comparative bioacoustics studies reported differences and similarities in acoustic parameters and frequency of emission between wild and domestic populations for dogs, cats, pigs, sheep and poultry [4–10].

Domestic chickens (*Gallus gallus domesticus*) are a well-known model in animal bioacoustics for their ability to produce, discriminate, and modulate different types of vocalizations [11–16]. Chicken calls are complex and functionally referential, meaning that they provide particular and precise information about the surrounding environment to their conspecifics [17]. Receivers respond to playbacks of food calls with increased anticipatory feeding behavior (i.e., greater inspection of the ground substrate), compared to contact or alarm calls playbacks [16]. Furthermore, males appear to adapt their food calls and communicate information about food quality to females [14]. In a similar fashion, behavioral responses to predators may vary with predator size: chickens exposed to large predators (high threat) produce relatively more aerial alarm calls, while more ground alarm calls are produced when birds are exposed to small predators (low threat) [15].

This complex communication system observed in domestic chickens has its roots in the selection pressures (e.g., the need to locate food sources, mate with optimal sexual partners, and hide from predators) undergone by their ancestor, the Red Junglefowl (*Gallus gallus*) [17, 18]. Interestingly, although the process of domestication is known to relax some of these pressures [19], domestic chickens and Red Junglefowl still maintain similar vocal repertoire and acoustic structure in their calls [20, 21]. Also, the behavioral responses to conspecific calls, such as alarm calls, seem to be innate: naïve domestic chicks spend longer time in tonic immobility (a fear reaction) following an exposure to adult alarm calls than to attraction calls [22].

Even though domestication did not appear to alter fundamental aspects of domestic chicken and Red Junglefowl acoustics, we can still hypothesize that these two breeds differ in their perception of different conspecific calls. Indeed, domestic chickens are more food motivated, less exploratory, and less fearful (in standardized behavioral tests, such as open field and predator tests) than Red Junglefowl [23–26]. On more complex cognitive tasks, such as spatial orientation and social learning tasks, we have recently shown that domestic chickens are more persistent and less flexible when facing an unreachable reward, but have an increased ability to use social information compared to Red Junglefowl [27, 28]. Combined, these results suggest that domestic and wild fowl do perceive their physical and social environment differently. To what extent these differences affect the perception of conspecific calls by these breeds is currently unknown.

From an applied perspective, the study of the impacts of conspecific calls on the behavior and welfare of farmed animal species may shed light on how animals are influenced by them [29]. For instance, distress and alarm calls are frequently witnessed in farming conditions due to various reasons, such as handling, transport, and slaughter, and may induce a poor welfare state on the listeners of such calls [29–31]. However, research on whether domestic animals interpret distress and alarm calls as such and react to them accordingly is still limited and may present some contradictions. For example, previous work on pigs revealed that individuals showed similar behavioral and physiological reactions following the playbacks of both conspecific distress calls and a neutral control sound [30], while recent research revealed a contrary pattern: pigs do discriminate negative and positive conspecific vocalizations (vocalizations made during social isolation and when in pairs, with access to food, water, and toys, respectively) [32].

Besides the established knowledge on chicken calls presented in the beginning of this section, discrepancies are also observed on more recent work: few behavioral and physiological differences were observed when comparing individuals’ reactions to neutral/positive (food call or trills of pleasure) and negative (alarm or distress calls) vocalizations, which suggests that domestic chickens may not be able to discriminate them [33–35]. These discrepancies may be related to the birds’ breed origin. While most of the fundamental work on chicken vocalizations used bantam chickens (a breed that was not subjected to intense artificial selection) as study subjects, the most recent applied studies used individuals from breeds selected for rapid growth and increased egg production. This intense artificial selection may have altered individuals’ perception and requires further investigation.

For a better understanding on whether and how domestication affected the perception of conspecific calls on chickens, in the present work, we compared the behavioral reaction (i.e., the time spent in vigilance behavior) of domestic and captive-born wild fowl to the playbacks of alarm and contentment calls. Alarm calls are produced following the approach of a predator [30], while contentment calls are produced during more positive contexts, such as feeding from a highly valued source [21]. Therefore, we expected the playback of alarm calls to cause a more pronounced state of vigilance in the animals, compared to the contentment calls (control). The playback tests were performed on four different breeds/lines. We first compared the behavioral reaction of domesticated White Leghorn (a breed selected for egg production) and unselected Red Junglefowl hens. Then, we compared the behavior of Red Junglefowl hens selected across 11 generations for high or low fear of humans, a proxy to investigate early effects of domestication [36–38]. We hypothesize that domestication would reduce the need of animals from certain breeds/lines (here, the White Leghorn and Red Junglefowl selected for low fear of humans) to react to alarm calls (through less vigilance behavior), since domesticated animals are mainly selected to thrive in a human-controlled environments, where predators are absent. On the contrary, unselected Red Junglefowl and those selected for high fear of humans were expected to react more strongly to the playback of conspecific alarm calls, due to high fearfulness of these individuals [24, 26, 39]. All breeds/lines were expected to react with more vigilance following the playbacks of alarm calls than the playbacks of contentment calls.

## Materials and methods

### Ethical statement

This study was conducted at the University of Linkoping, Sweden, in March-April 2022. All applicable international, national, and/or institutional guidelines for the care and the use of animals followed the 1964 Helsinki Declaration and its later amendments or comparable ethical standards. The study was approved by the Linköping Council for Ethical Licensing of Animal Experiments (license number 14916–2018).

### Animals and housing

Hens from two different breeds were used in this experiment: the domesticated laying breed White Leghorn (WL), and the wild breed Red Junglefowl (RJF). Females were chosen over males, as they are known to be more food motivated [40], which was more appropriate for our experimental set-up.

WL parental birds (males and females) were from a non-commercial population, initially obtained from the Swedish University of Agricultural Sciences in 1998, while RJF originated from a zoo population in the same year. Since then, both breeds are being kept in our research facilities and have being bred over multiple generations (for further details concerning both the domesticated and captive wild population, see [23]).

Starting from an outbred group of RJF, based on two different zoo populations, we selected, over 11 generations, birds for high and low fear of humans (RJF HF and RJF LF, respectively). At 12 weeks-old, a fear-of-human test was performed on individuals of each generation. The test consisted on measuring the individuals’ reaction to a standardized human approach. Birds were scored on a scale going from 1 (most relaxed) to 5 (most fearful). Further details on the breeding and selection program can be found in [41].

A full description of the husbandry procedures is described in [23, 28, 42, 43]. Briefly, eggs from both breeds/lines were incubated and hatched under the same conditions at the University of Linköping, Sweden (Unselected RJF hatched on October 2020; WL hatched in December 2020; RJF HF and LF hatched in February 2021). From day 1 to day 17 of incubation, the incubator ambient settings were of 37.8°C, and 55% of relative humidity. On day 18, and until hatching, eggs were placed in a hatcher with the following settings: 37.5°C and 65% relative humidity.

On the day after hatching, the chicks were taken out of the incubator, weighed, and individually identified with wing tags. Up to 5 weeks of age, chicks were kept in mixed-sex groups in indoor pens (Unselected RJF: 99 individuals; WL: 29 individuals; RJF HF and LF: 75 individuals). All pens were supplied with sawdust, a heating roof, a feeder, and a water bell. The size of the pens was adjusted (~ 0.5 m^2^–3 m^2^) with increasing age, following chick growth. The pens were cleaned once a week and had a 12-hour light/dark schedule. At 5 weeks of age, chicks were moved to another research facility (the Wood-Gush chicken facility), approximatively 10 km away from Linköping. Birds were separated by sex and kept in an indoor multi-tier pen (3 × 3 × 3 m^3^), with perches and nest box (Unselected RJF: 56 females WL: 19 females RJF HF and LF: 35 females). Also, birds had free access to an additional, fully enclosed outdoor area (3 m^2^). From their arrival at the research facility, WL and unselected RJF were kept in separate pens, while HF and LF RJF lines were kept in the same pen. Birds in the research facility had visual, olfactory, and auditory contact with multiple conspecifics (males and females) from other pens.

### Call collection and stimuli preparation

**Fig 1:**
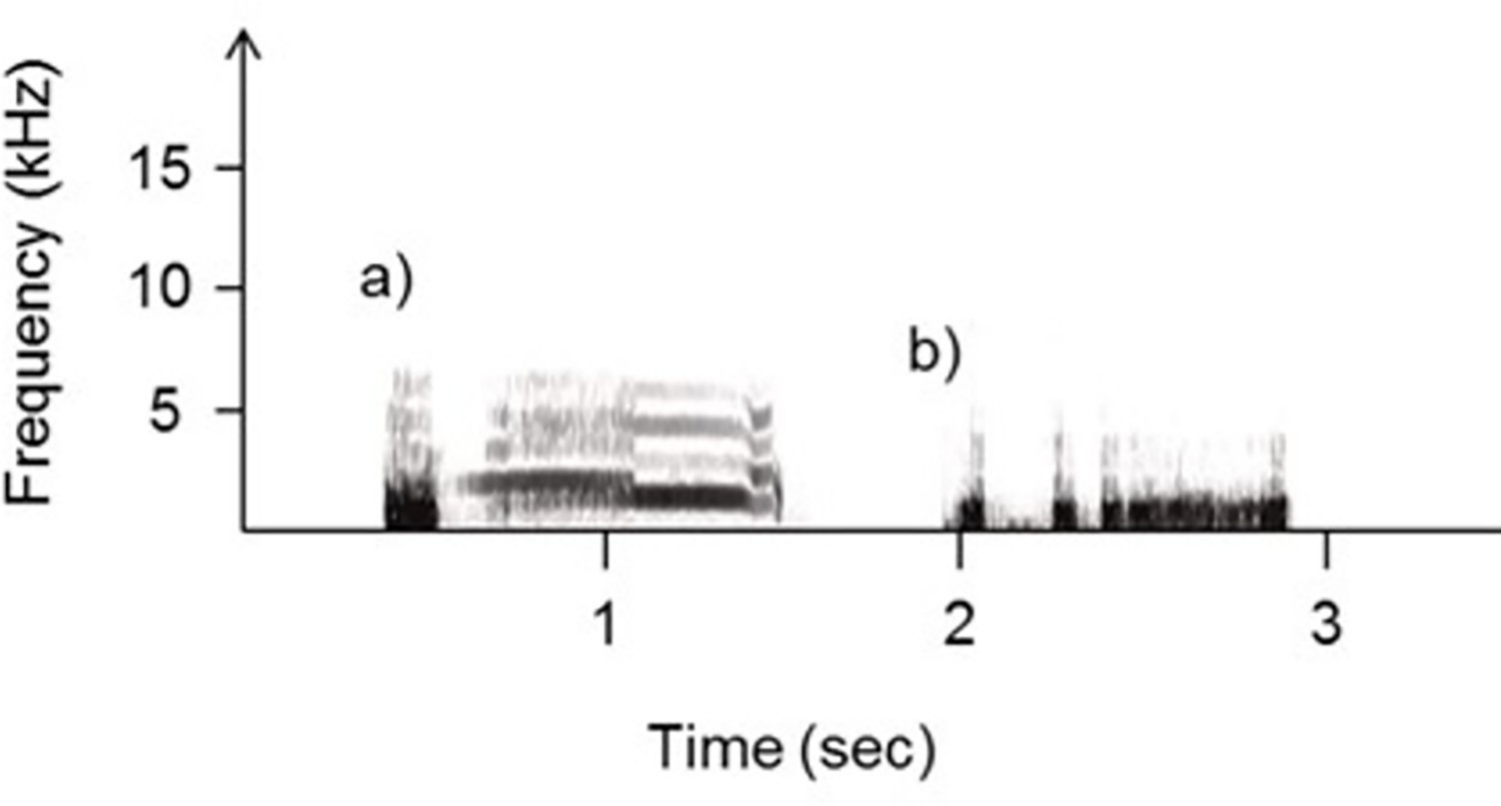
Spectrograms of sound treatments played to chickens. a) alarm call (test playback) and b) contentment call (control playback).

Alarm and contentment chicken calls were obtained from different audio resources (n = 3 for each type of treatments). These recordings were chosen for their high signal-to-background ratio and low levels of background noise. We kept the total sound duration similar between alarm call playbacks and contentment call playbacks (mean duration of each alarm or contentment call is ~1 second, Fig 1A and 1B). The contentment call and the alarm call were repeated three times per playback sequence. Calls were separated by 1.4 sec (natural break between alarm calls: 1.3 sec, n = 10; natural break between contentment calls: 1.6 sec, n = 3). Playback sequences were prepared using the program Avisoft-SASLab, saved as WAV files, and transferred to a loudspeaker (Zealot S1) for playback.

### Habituation period and playback experiments

From one to two weeks before the beginning of the playback experiments, 16 WL (One year and three-months-old), 16 RJF (One year and six-months-old), 12 RJF HF, and 16 RJF LF (One year-old) hens were selected for testing, captured, and identified with colored plastic leg rings. All individuals were naïve to playback experiments. Experiments on WL/Unselected RJF and RJF HF/RJF LF were performed two weeks apart. In order to reduce daily capture stress, hens were moved and kept in randomly-defined pairs of the same breed/line in battery cages (0.6 × 0.4 × 0.4 m) with *ad libitum* water and food, perch, nest box and dust bath on the day the experiment started.

Prior to the first test day, hens were allowed to habituate to the test situation in one of three adjacent wooden pens (0.7 × 0.7 m) built in a calm testing room. Hens were placed in the testing pens in groups of four (always the same two pairs from the same breed/line) and left undisturbed for 40 min per day, over three days. The floors of the testing pens were covered with sawdust mixed with common chicken feed and sweet corn. This habituation procedure was needed to ensure that any changes in birds’ behavior was due to the playback treatment, and not attributed to the fear of an exposition to the new environment.

During the two subsequent testing days (between 9am– 1pm), pairs of hens from the same breed/line were exposed to one playback stimulus per day (alarm calls or contentment calls, repeated three times/playback sequence). Only one pair was tested at a time. To avoid playback habituation, a minimum period of one day (24h) was left between testing days and each pair was randomly assigned to a stimuli sequence (alarm call–contentment call or contentment call–alarm call) out of nine possible combinations (based on three alarm calls and three contentment calls). During the second testing day, the same pairs were exposed to the playback stimulus which they did not receive the previous first testing day. Testing order of the pairs was balanced between the breeds/lines (WL vs. RJF and RJF HF vs. RJF LF). Within the testing time, half of the pairs tested earlier in the first testing day, were tested later in the second testing day, and vice-versa.

Each pair of bird was gently captured in their cages and placed in the testing pen while lights were off. The test started when lights were switched on. Birds were left undisturbed for 5 min in the testing pen and, similar to during the habituation phase, had access to sawdust, food, and sweet corn. By the end of the 5-min period, the playback calls were broadcast. Playbacks were presented at a distance of 2 m away from the birds from the loudspeaker that was at ~ 0.6 m from the ground and oriented towards the individuals. To prevent differences in the intensity of the response of the bird, all calls were broadcast with the same intensity. Following previous studies [16, 33, 34], calls were played at ~70 dB (A) (measured using a Tadeto SL720 sound level meter, at the bird level). Birds were kept in the testing pen for 2 min following the broadcast of the playback stimulus, and then brought back to their cages. Hens were put back in their home pens as soon the experiment was over.

The whole experiment was monitored and recorded directly by an experimenter outside of the view of the tested animals, using a digital video camera recorder connected to a monitor.

### Analysis of videorecorded behavioral responses

Recordings of behaviors of the birds began 60 sec before playbacks were initiated, continued during the playback, and for 60 sec after the beginning of the playback [16]. From video analysis, the variable scored was the time birds spent on vigilance behavior (i.e., birds lift up their head and scan the environment with little or no body movement. Birds could be in a standing or sitting position). All recorded videos were analyzed by the same experimenter (VHBF). Videos were muted so that the experimenter was not aware of which playback stimulus was being played. To control for the reliability of vigilance scoring, a subset of these playbacks (~12%) was analysed by a second experimenter (MD). A Spearman correlation revealed a high concordance between observers (r_s_ = 0.89, p < 0.001, n = 14).

### Statistics

All statistical analyses were performed using IBM SPSS 21. Since birds were tested in pairs, and their behavior are known to be highly synchronized and modulated by the presence of other conspecifics [12, 27, 44, 45], in order to reduce possible social influences, the vigilance behavior of each pair was averaged (only one value per pair and time condition, i.e., before or after the playback) [46]. Moreover, since data did not meet the assumptions of parametric tests, only non-parametric analyses were used.

To ensure no differences were present before the playback stimuli was broadcast, we first compared, using Mann-Whitney tests, whether birds’ vigilance behavior differed before the two different playback stimuli. This first analysis was done within each day and within each breed/line. Then, we compared the breeds/lines between them (RJF vs. WL and RJF HF vs. RJF LF) to check for different vigilance patterns before the playback over testing days 1 and 2, without taking into account the type of playback call. Finally, we checked, using a Wilcoxon test, whether intragroup vigilance before the playback differed between days (for each line and without taking into account the type of playback call).

Following the same procedure, the previous analyses were repeated to investigate the vigilance of birds after the playback stimuli. Additionally, we compared the breeds/lines between them (Mann-Whitney, RJF vs. WL and RJF HF vs. RJF LF) to check for different vigilance patterns after the playback stimuli, without taking into account the testing day

If one individual in the pair was highly vigilant before the broadcast of the playback stimulus (time spent in vigilance behavior > relaxed behavior, such as foraging), its data points were discarded, and only the vigilance behavior of its companion was accounted for the pair (WL vs. RJF day 1, n = 2 WL and 2 RJF hens discarded; day 2, n = 3 WL and 2 RJF hens discarded; RJF HF vs. LF day 1, n = 1 RJF HF and 1 RJF LF hens discarded; day 2, n = 1 RJF HF and 1 RJF LF hens discarded). If both individuals in one pair were highly vigilant before the broadcast of the playback stimulus, their data points were discarded for the two testing days (WL vs. RJF day 1, n = 1 WL pair discarded; RJF HF vs. LF day 1, n = 1 RJF LF pair discarded; day 2, n = 1 RJF LF pair discarded). One WL hen died during tests (for reasons not related to the experiment), and the data points from the whole pair was therefore discarded. Our final N comprised 6 pairs of WL hens, 8 pairs of unselected RJF, 6 pairs of RJF selected for high fear of humans, and 6 pairs of RJF selected for low fear of humans. Statistical significance was accepted at p ≤ 0.05 and tendencies at p ≤ 0.1.

## Results

The time spent in vigilance behavior (presented as raw means ± SD), before and after the playback of contentment and alarm calls, on each of the two testing days was compiled, for all breeds/lines, in Table 1.

**Table 1:** Time spent in vigilance behavior (in seconds), before and after the playback of contentment and alarm playback calls, on each of the two testing days (Day 1 and Day 2), for domestic White Leghorn (WL) and unselected Red Junglefowl (RJF) hens, and for Red Junglefowl hens selected for High (HF) and Low fear (LF) of humans.

	Time spent in vigilance behavior (in seconds)							
Breed/Line	Alarm call				Contentment call			
	Day 1		Day 2		Day 1		Day 2	
	Before	After	Before	After	Before	After	Before	After
RJF	2,3 ± 2,8	21,3 ± 25,8	2,3 ± 2,8	26,5 ± 17,2	3,6 ± 5,4	31 ± 30,4	1,2 ± 2,5	15,8 ± 12,5
WL	5,3 ± 1,2	44,5 ± 26,8	8,8 ± 6,5	45,8 ± 20,3	6,6 ± 5,8	60 ± 0	7 ± 12,1	23,6 ± 16,7
RJF HF	3,1 ± 3,5	47 ± 14	7,5 ± 12,9	31,8 ± 24,4	5 ± 8,6	60 ± 0	7,8 ± 1,1	38,8 ± 22,4
RJF LF	10,5 ± 3,2	52,8 ± 12,9	7,7 ± 10,9	17,2 ± 2,4	1,5 ± 2,1	53,7 ± 8,8	4 ± 6,7	14,2 ± 8,4

Mean ± SD are given (n_WL_ = 6 pairs, n_RJF_ = 8 pairs, n_HF_ = 6 pairs, n_LF_ = 6 pairs).

### White Leghorn vs. Red Junglefowl breeds

#### Before the playback broadcast

On both testing days 1 and 2, and for each breed, vigilance before the playback did not differ between alarm and contentment calls (Mann-Whitney, Day 1 RJF: U = 8, p = 1; Day 2 RJF: U = 6, p = 0.71; Day 1 WL: U = 3, p = 0.7; Day 2 WL: U = 3, p = 0.6). Vigilance before the playback on day 1 and day 2 did not differ between the breeds (Mann-Whitney test, Day 1: U = 13, p = 0.161; Day 2: U = 14, p = 0.196). Finally, vigilance before the broadcast of the playback stimuli did not vary between the days (Wilcoxon, D1 vs. D2 WL: Z = -0.314, p = 0.844; D1 vs. D2 RJF: Z = -0.339, p = 0.797).

#### After the playback broadcast

**Fig 2:**
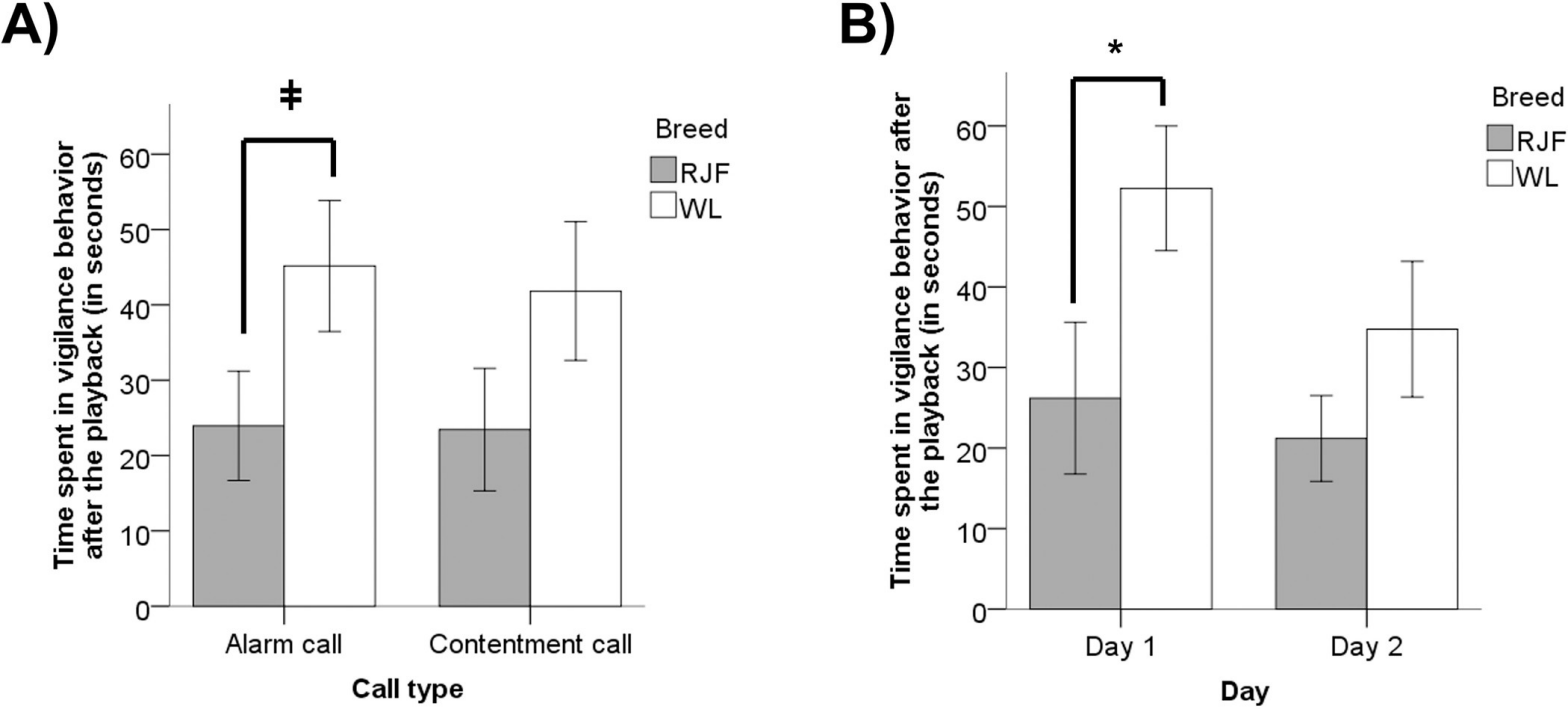
Time spent in vigilance behavior (in seconds) for White Leghorn (WL) and Red Junglefowl (RJF) hens, A) after the playbacks of contentment and alarm calls, and B) after the playbacks and over testing days (Day 1 and 2, without taking into account the playback call type). Mean ± SD are given. (n_WL_ = 6 pairs, n_RJF_ = 8 pairs).

Within each day and within breed, vigilance after the playback did not differ between alarm and contentment calls (Mann-Whitney, Day 1 RJF: U = 7.5, p = 0.97; Day 2 RJF: U = 4.5, p = 0.37; Day 1 WL: U = 3, p = 1; Day 2 WL: U = 1, p = 0.2). Without taking into consideration the testing day, WL hens tended to react with more vigilance than RJF after the alarm call playback was broadcast (Mann-Whitney, U = 9.5, p = 0.062, Fig 2A), but no vigilance differences were found after the broadcast of contentment calls (Mann-Whitney, U = 11.50, p = 0.11, Fig 2A). Furthermore, within each breed, there were no differences in vigilance behavior following alarm and contentment calls (WL: Z = -0.135, p = 1; RJF: Z = -0.35, p = 0.398).

Without taking into account the playback call type, on day 1, WL were significantly more vigilant after the playback, than RJF hens (Mann-Whitney, U = 8, p = 0.03, Fig 2B), while vigilance levels did not differ between breeds after the playback on day 2 (Mann-Whitney, U = 13, p = 0.17, Fig 2B). Vigilance after the broadcast of the playback stimuli did not vary between the days (Wilcoxon, D1 vs. D2 WL: Z = -1.753, p = 0.125; D1 vs. D2 RJF: Z = -0.911, p = 0.398).

### High fear RJF vs. Low fear RJF lines

#### Before the playback broadcast

On both testing days 1 and 2, and for the two lines, vigilance before the playback did not differ between alarm and contentment calls (Mann-Whitney, Day 1 HF: U = 0, p = 0.13; Day 2 HF: U = 3, p = 0.8; Day 1 LF: U = 4, p = 1; Day 2 LF: U = 3, p = 0.8). Vigilance before the playback on day 1 and day 2 did not differ between the lines (Mann-Whitney test, Day 1: U = 11.5, p = 0.327; Day 2: U = 15, p = 0.675). Also, no difference in vigilance behavior before the playback between day 1 or day 2 was found for the two breeds (Wilcoxon, RJF HF: Z = -0.944, p = 0.438; RJF LF: Z = -0.734, p = 0.563).

#### After the playback broadcast

Within each day and within line, vigilance after the playback did not differ between alarm and contentment calls (Mann-Whitney, Day 1 RJF HF: U = 4, p = 1; Day 2 RJF HF: U = 2, p = 0.53; Day 1 RJF LF: U = 0, p = 0.1; Day 2 RJF LF: U = 4, p = 1). No differences between the lines were found after the alarm and contentment calls, even when disregarding testing days (Mann-Whitney, Alarm calls: U = 15, p = 0.699; Contentment calls: U = 7.5; p = 0.102). Similar to the unselected parental breeds, there was no difference at the intragroup level in vigilance behavior following alarm and contentment calls for the two lines (Wilcoxon, RJF HF: Z = -0.944, p = 0.438; RJF LF: Z = -0.943, p = 0.398).

**Fig 3:**
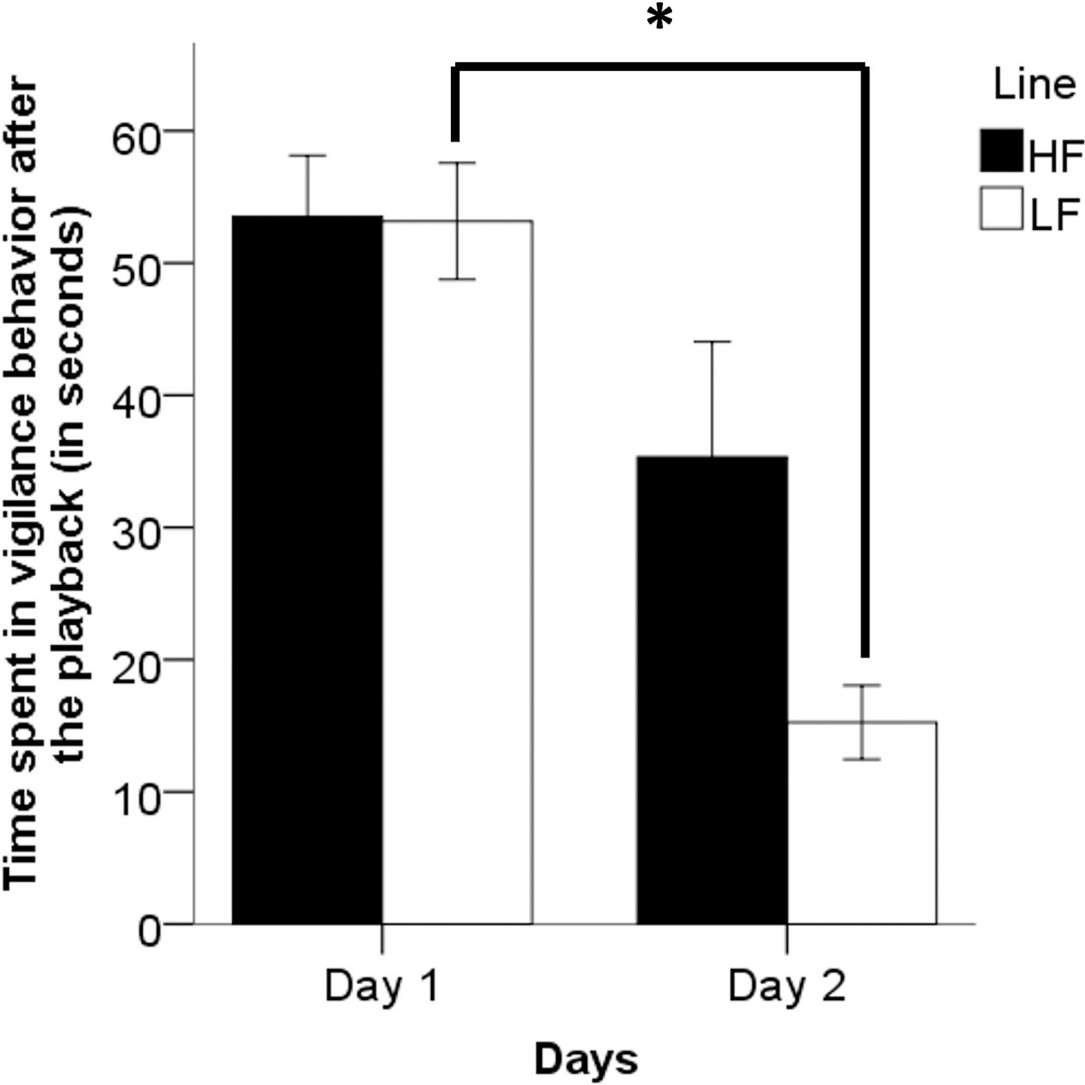
Time spent in vigilance behavior (in seconds), after the playbacks and over testing days (Day 1 and 2, without taking into account the playback call type), for Red Junglefowl hens selected for High (HF) and Low fear (LF) of humans. Mean ± SD are given. (n_High_ = 6 pairs, n_Low_ = 6 pairs).

Without taking into account the playback call type, vigilance after the playback on day 1 and day 2 did not differ between the lines (Day 1: U = 17.5, p = 1; Day 2: U = 8, p = 0.132). However, LF birds diminished their vigilance levels between days after the playback (Wilcoxon, Z = -2.201, p = 0.031, Fig 3), but this was not the case for HF birds (Wilcoxon, Z = -1.483, p = 0.188).

## Discussion

In the current work, we investigated whether and how domestication influenced the behavioral reaction (i.e., vigilance behavior) of domestic and captive-born wild fowl to two contrasting intraspecific calls (contentment call vs. alarm call). On that purpose, we compared the vigilance reaction of White Leghorn hens, a breed strongly selected for egg production, to that of Red Junglefowl hens, the ancestor of all domestic chickens. Additionally, two selected lines of RJF hens that emulate the domestication process were also compared (individuals selected for high vs. low fear of humans). Contrary to our expectations, no breed/line reacted accordingly to the calls, as the increase in vigilance behavior after the playback calls was similar for both contentment and alarm calls. Although no call discrimination differences were found, breeds did differ on how they reacted/habituated to the calls. Overall, WL were more vigilant than RJF, and birds from the RJF LF line decreased their vigilance over testing days, while this was not the case for the RJF HF line. These results agree, to a certain extent, with previous studies on that domestication and selection for low fear of humans (a major feature of domestication) alters how birds cope and habituate to frightening stimuli [36, 39, 47]. Due to the small sample size tested, the interpretation of our results requires caution, multiple alternative interpretations are discussed below.

The failure of the tested birds to discriminate between the two tested intraspecific calls is in stark contrast to previous fundamental work on chicken vocalizations, which shows that chicken possess abilities in discriminating different types of calls [11–16, 48]. Although speculative, there are some non-mutually exclusive explanations that should be taken into account when interpreting and comparing our results to previous ones. First, it is necessary to consider the early and late environments where individuals were reared. Most of the fundamental work on chicken vocalizations were done in bantam chickens, an ornamental breed that was not selected for any production purposes (such as high egg yield or rapid growth) and kept many behavioral features similar to that of wild-born RJF [15, 16]. Although this information is rarely given in previous studies, it is highly likely that chicks from bantam breeds had access to maternal care when young [15], and that outside of the experimental condition, birds had access to naturalistic conditions, on large open-air outdoor enclosures [15, 49]. Combined, these different and rich experiences may have allowed birds to better learn about their physical and social environment and to have a fine-tuned panel of behavioral responses that were adapted to a variety of situations, which is in total opposition of what was experienced by birds tested in the current study (i.e., individuals were hatched in an incubator, reared without maternal care, and had access to a small enclosed outdoor area). Indeed, animals born in captivity show impaired anti-predator behavior [46, 50–52], evidencing that the absence of species-specific meaningful experience may impact how individuals interpret certain stimuli. It is known that adaptation to laboratory conditions may alter how animals cognitively perceive and react to their environment, making domestic and captive-born wild individuals to behave alike, when compared to individuals born and raised in the wild [53]. Additional, exploratory analyses comparing the vigilance behavior of parental and selected (HF and LF) RJF support these results: no significant differences between the three RJF groups for any of our variables (Kruskal-Wallis test, all p > 0.05). Since the tested birds were reared in the same commercial-like conditions, they may have developed a similar and undiscriminated vigilance response to the auditory stimuli investigated here.

Previous studies on the reaction of naive chicks to alarm calls suggest that fowl discrimination of intraspecific calls may be innate [22]. If that was the case, an appropriate behavior following the playback calls (i.e., increased vigilance after alarm calls compared to contentment calls) could be expected even in the absence of an experience with more naturalistic situations, as mentioned previously. However, it is important to mention that, it was not uncommon for the tested birds to produce loud alarm calls (up to 100 dB (A)) when a human suddenly entered the home pen room (VHBF, personal observation). Therefore, we cannot disregard the possibility that birds may have learned over time that reacting to those calls was not useful to thrive in their environment [54, 55], where predators are absent and food is accessible in an *ad libitum* manner.

Another reason for the absence of call discrimination among the tested birds is that the calls broadcast came from unknown individuals [30]. This new situation may have caused the tested birds to be more attentive to their surroundings, to better assimilate the calls of the “new” conspecifics. In a similar fashion, while the alarm calls may have generated anti-predator vigilance behaviors (besides the fact that it comes from a new individual), the contentment calls may also have signaled the arrival of a new individual and as such caused a more “territorial” vigilance. However, this hypothesis is unlikely to explain our results since these birds were constantly exposed to new individuals that arrived at our facilities. Therefore, they were expected to be habituated to hear different calls from different individuals. Furthermore, recent research shows no evidence for emotional empathy in chickens observing familiar adult conspecifics undergoing a mild stress [56]. The emotions expressed during the less positive alarm calls and more positive contentment calls were, probably, not sufficient to elicit a differential response in the tested individuals. More research is necessary to understand how fowl reared under commercial-like conditions interpret the calls from known and unknown individuals, and how this impact their behavior.

Interestingly, the vigilance behavior of our parental breeds did differ between WL and RJF hens, with WL being more vigilant on day 1 and more vigilant after an alarm call than RJF. These data suggest that domestication have altered how these breeds cope to frightening stimuli. Indeed, it is known that white strains of domestic hens have a proactive coping style [57], while RJF cope with stress in a more reactive manner [24, 58]. Due to their proactive characteristics, it is possible that WL hens habituated quicker to the silent testing arenas (during the habituation phase, for example) and were more disturbed to the sudden playback compared to RJF. It is also known that WL are less motivated to express foraging behaviors [25], so their need to resume a normal behavior (such as foraging) following the playback may be less important, compared to their RJF counterparts. Another reason for these results could be that, although domestic chickens and RJF are known to have a similar vocal repertoire and acoustic structure of their calls, the playback calls used here (from domestic birds) could be more relevant for WL chickens and to a lesser extent for RJF [20, 21]. Future studies must take into account all of these interfering variables in order to disentangle their effects and better understand the animals’ behavioral responses after playbacks.

Vigilance behavior decreased over days for RJF LF, but not for RJF HF. These results agree with the hypothesis that selection for low fear of humans cause animals to habituate quicker to fearful stimuli. Indeed, RJF LF habituated quicker to the onset of a frightening flash light and showed less fear reactions between the first and the second testing, compared to RJF HF [36, 47]. This habituation process may be adaptative for individuals to thrive in human-controlled environments, where frightening events (for example, sudden noises or light, and human presence) occur on a daily basis.

To conclude, our results suggest that birds under commercial-like conditions appear, based on their vigilance behavior, unable to discriminate between contrasting alarms and contentment calls. Our results also suggest that domestication and selection for low fear of humans may have altered how birds react/habituate to startle stimuli. Beyond the fundamental interest of understanding how domestication affects the perception of individuals, our results should also be taken from a more applied perspective: farmed animals may interpret and be affected by the vocalizations of their conspecifics in unexpected ways that warrant further investigation.

## Supporting information

S1 Data (XLSX)
